# cGAS/STING Pathway in Cancer: Jekyll and Hyde Story of Cancer Immune Response

**DOI:** 10.3390/ijms18112456

**Published:** 2017-11-18

**Authors:** Debojit Bose

**Affiliations:** Laboratory of RNA Biochemistry, Institute of Chemistry and Biochemistry, Freie Universität Berlin, Takustrasse 6, 14195 Berlin, Germany; debojit.bose@fu-berlin.de

**Keywords:** cGAS, STING, Cyclic guanosine monophosphate–adenosine monophosphate (cGAMP), cancer, immunity, cancer immunotherapy

## Abstract

The last two decades have witnessed enormous growth in the field of cancer immunity. Mechanistic insights of cancer immunoediting have not only enhanced our understanding but also paved the way to target and/or harness the innate immune system to combat cancer, called cancer immunotherapy. Cyclic GMP-AMP synthase (cGAS)/Stimulator of interferon genes(STING) pathway has recently emerged as nodal player in cancer immunity and is currently being explored as potential therapeutic target. Although therapeutic activation of this pathway has shown promising anti-tumor effects in vivo, evidence also indicates the role of this pathway in inflammation mediated carcinogenesis. This review highlights our current understanding of cGAS/STING pathway in cancer, its therapeutic targeting and potential alternate approaches to target this pathway. Optimal therapeutic targeting and artificial tunability of this pathway still demand in depth understanding of cGAS/STING pathway regulation and homeostasis.

## 1. Introduction

The cross-talk between cancer and the immune system was reported first in the 1960s [1,2], but the relative inefficacy of naturally occurring immune responses coupled to lack of understanding of the underlying molecular mechanisms posed a major challenge in this field. The immune system can selectively recognize and kill cancer cells, a process called tumor immunosurveillance. As a counter strategy, cancer cells have evolved to bypass this process and use the immune system to promote tumorigenesis. This dual role of the immune system in both suppressing and promoting cancer, called cancer immunoediting, poses a challenge from a therapeutic perspective [1]. With the advent of recent technologies, accompanied with a better understanding of the molecular mechanisms involved, the last two decades have seen tremendous growth in the field of cancer immunotherapy.

The cGAS/STING pathway was initially described to play a crucial role in antimicrobial immune response. Following activation by aberrant cytosolic DNA, the enzyme cGAS produces the mammalian 2′,3′-cGAMP, which in turn activates STING protein and thereby leads to production of Type I interferon (IFN) and other pro-inflammatory cytokines that boost the immune response. Presence of microbial DNA in the cytosol seemed to be the major activator of cGAS [3]. Cyclic di-nucleotides (CDNs) produced by certain bacteria were also shown to activate some isoforms of human and mice STING by direct binding [4,5,6,7]. According to recent studies, self-DNA leaked from nucleus or mitochondria, probably followed by cell division, DNA damage and/or autophagy, can also activate this pathway, leading to pathophysiological outcomes [8,9].

Apart from its role in protecting the host from a variety of pathogenic attacks, cGAS/STING pathway also plays a crucial role in cancer. While activation of cGAS/STING pathway has mostly been reported to produce anticancer effects [10,11,12,13], evidence also suggests that it is implicated in carcinogenesis via self-DNA induced autoinflammation [14,15,16,17,18]. Here, recent understanding of the role of cGAS/STING pathway in cancer and its therapeutic modulation has been reviewed, with an aim to emphasize the point that a better understanding and artificial tunability is required for optimal targeting of this pathway as a potential cancer immune therapy. Potential strategies to discover small molecule modulators of the cGAS/STING pathway from a therapeutic perspective have also been discussed.

## 2. Type I IFN, Immune Response and Cancer

Cancer specific antigens and endogenous expansion of CD8+ T cells have been discovered in many cancers. Clinical studies indicate spontaneous T cell priming, immune infiltration of T cell recruiting cytokines, and Type I IFN response into tumor sites; a phenomenon called T cell inflamed microenvironment [19]. Harlin et al. reported a strong correlation between tumor infiltrating CD8+ T cells and chemokine expression in metastatic melanomas. In subset of melanoma metastasis, it was suggested that reduced critical chemokine expressions is a key factor in limiting activated T cell migration and, thereby, effective anti-cancer response [20]. In anti-cancer immune response, the maturation and activation of antigen-presenting dendritic cells (DCs) is a critical step for activating the T-cell response to kill cancer cells. This is blocked by tumor cell-derived cytokines such as IL-10 and TGFβ. Thus, co-stimulatory inflammatory signals are immensely important for T cell activation. In context of microbial infection this is often mediated by Toll-like receptor (TLR) stimulation. Very little was known about the mechanism in sterile state, until recent gene expression profiling data indicated Type I IFN response to be a key player in activating T-cell response. Ablation of Type I IFN response in vivo, either by IFN receptor (IFNAR) deletion or treatment with antibody, was shown to enhance chemically induced tumor formation. This also showed weaker transplanted immunogenic tumor reduction than wild type (WT) mice, highlighting the importance of Type I IFN in spontaneous tumor rejection [21]. The carcinogen methylcholanthrene treatment showed enhanced tumor production in IFNAR−/− mice [21,22]. DCs were shown to be the main players in stimulating Type I IFN signaling [23,24]. The induction of tumor-specific CD8+ T cells, leading to immune rejection of tumors, was predominantly mediated by Type I IFN production in dendritic cells (DCs) [25]. In summary, initial activation of anti-tumor innate immune response depends on Type I IFN production by DCs which eventually helps in CD8+ T cell cross priming, followed by tumor cell killing. Upon radiotherapy, increased intratumoral Type I IFN production was found. This was associated with increased cross priming potential of tumor infiltrating DCs and could be eliminated by removing IFNAR [26]. Similar dependency on Type I IFN in anti-tumor CD8+ T cell induction was also reported in context of tumor cell therapy. Type I IFN response was also shown to have anti-angiogenic response [27].

Therefore, Type I IFN seems to be nodal player in eliciting an effective anti-tumor immunity by acting as a bridge between innate and adaptive immunity. A better molecular understanding of Type I IFN production and regulation will be helpful in basic and therapeutic perspective.

## 3. cGAS/STING Pathway Induced Type I IFN Production and Cancer Immunity

**Figure 1:**
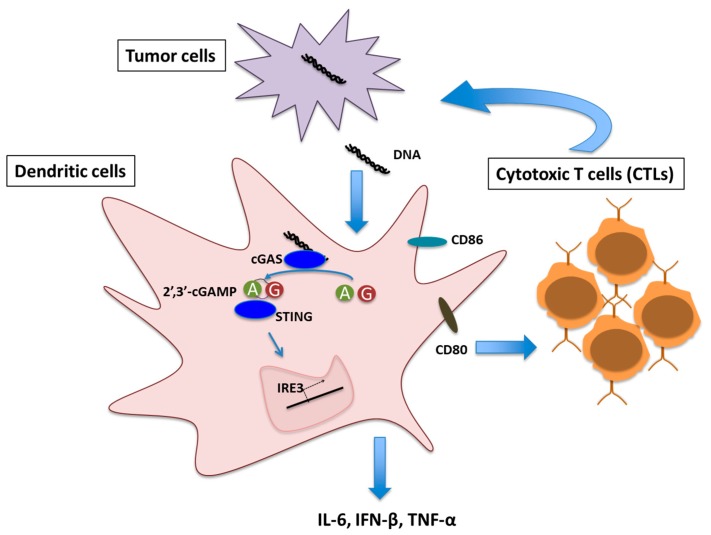
Schematic representation of Cyclic GMP-AMP synthase (cGAS)/Stimulator of interferon genes(STING) signaling mediated T cell priming in tumor microenvironment. A and G denotes Adenosine monophosphate (AMP) and Guanosine monophosphate (GMP) respectively.

Given the role of Type I IFN in optimal T cell priming against tumor, the obvious mechanistic question was to identify the molecular pathway(s) that trigger IFN production in DCs in cancerous conditions. Recent evidence suggests cGAS/STING signaling as one of the key pathways in this context. Transplantable mouse tumor model study showed defective T cell priming against tumor antigen in STING−/− and IRF3−/− mice [12]. In a colon cancer model, increased phosphorylation of NF-κB and STAT3 leading to transcriptional suppression of pro-inflammatory cytokines IL-6 and keratinocyte chemoattractant (KC) was observed in STING−/− mice [28]. Increasing evidence indicates the presence of cytoplasmic DNA in cancer cells that can induce IFN response via cGAS/STING pathway [13,29]. Damaged genomic DNA caused by carcinogens like DMBA, cisplatin, etoposide or radiation [13,30,31] and mitochondrial DNA leakage [32] has been shown to be the primal sources of the cytoplasmic DNA in cancer cells that can potentially activate cGAS/STING mediated immune response. DNA damage induced micronuclei in cancer cells also stimulate cGAS/STING pathway following membrane damage [33]. These clearly indicate a strong link between DNA damage response and cGAS/STING pathway mediated cancer immune response. DNA fragments derived from cancer cells, present in the tumor microenvironment, were shown to be taken up by DCs. This DNA fragments activated cGAS/STING pathway, inducing Type I IFN response and thereby activating DC maturation. Matured DCs in turn stimulated CD8+ T cell priming (Figure 1) [13].

Gliomas, when induced de novo in STING−/− mice, showed shorter survival associated with increased immune-suppressor cells and decreased IFN producing CD8+ T cells in a tumor microenvironment compared to wild type mice. Lower expression of Type I IFN and Type I IFN-inducible Interferon-stimulated gene 54 (ISG54) was also reported in STING−/− mice compared to the wild type mice. Intratumoral delivery of the STING agonist, cyclic di-GMP, improved survival via enhanced Type I IFN production [34,35]. STING signaling is severely suppressed in colorectal carcinoma associated with obstructed anti-tumor T cell priming and Type I IFNs; a strategy of tumor cells to evade the immune surveillance pathway [36]. In colitis associated colorectal cancer model, STING was shown to have a protective role via regulation of intestinal inflammation. STING−/− mice were prone to tumor formation due to excessive colon inflammation [28]. STING−/− mice were also shown to be prone to colitis associated cancer, induced by DNA damaging agents. STING constitutes a crucial response to intestinal damage and is essential for stimulating tissue repair pathways to prevent tumorigenesis [28]. A crucial role of STING pathway in response to cryoablation was shown in OVA model. The resulting Type I IFN expression enhanced DC functionality, resulting in clonal expansion, polyfunctionality and memory formation of tumor-specific CD8+ T cells [37]. In line with the previous knowledge of radiation induced cellular stress and excessive DNA damage, this report has also indicated cGAS mediated sensing of irradiated-tumor cells by DCs. cGAS/STING-dependent cytosolic DNA sensing pathway in DCs was shown to be essential for Type I IFN induction after radiation, which determines the radiation-mediated adaptive immune responses [12]. Macrophages were also found to be key players in cGAS/STING mediated cancer immunity [28], indicating the involvement of other antigen presenting cells (APCs) beyond DC-T cell axis. Furthermore, recent evidence suggests tight regulation of cGAS/STING signaling in T cells [38,39]. T cells from patients carrying constitutive active STING mutant (TMEM173) showed decreased cell proliferation and IL-2 production, cell cycle arrest and increased apoptosis [38]. In murine model, sustained activation of STING via STING agonists showed enhanced ER stress and activation of cell death pathways [39]. Therefore, STING activation and homeostasis seems to be tightly regulated in a cell type specific manner. Hence, further investigation in the context may indicate the predisposition of different cell or cancer types to cGAS/STING mediated anti-tumor immunity.

Not surprisingly, cancer cells have evolved defense mechanisms. In human colon cancer model, cGAS and STING promoter hypomethylation was shown to reduce expression of cGAS and STING. This, in turn, inhibited DNA damage dependent cytokine production, allowing the cancer cells to bypass the immune surveillance [36]. Similar epigenetic silencing of cGAS and STING has also enabled melanoma cells to escape immune surveillance upon DNA damage [40].

## 4. Therapeutic Targeting of cGAS/STING Pathway in Cancer

Given the suppressed Type I IFN response in non-T cell inflamed tumors, boosting robust immune signaling in tumor microenvironment has the potential to enhance cross-priming of tumor specific CD8+ T cells [41]. With the growing evidence and understanding of the role of cGAS/STING pathway in facilitating anti-tumor immunity, recent efforts are targeting to modulate this cGAS/STING pathway in context of cancer immune therapy.

### 4.1. Targeting with Small Molecule and CDNs

The small molecule 5,6-dimethyllxanthenone-4-acetic acid (DMXAA) initially showed potent anti-tumor activity in various mouse models [42,43], but they completely failed in phase III clinical trial in non-small cell lung cancer when combined with chemotherapy. Recent studies have shown DMXAA to be a direct mouse STING activator and the fact that it cannot interact with human STING explains the lack of clinical activity in human [44]. This has also necessitated the screening for small molecule activators of human STING. CDNs having the potential to directly activate STING have thus been explored for their anti-cancer immune potential. Cyclic di-GMP (c-di-GMP) was shown to enhance the immunogenicity and anti-tumor effect of a peptide vaccine, TriVax, in mouse B16 melanoma model [45]. c-di-GMP was shown to improve vaccination against metastatic breast cancer [45]. While low doses of c-di-GMP provided strong adjuvant effects in vaccinations, high c-di-GMP dosage activated caspase-3 causing direct tumor cells killing [46]. Enhanced survival of glioma-bearing mice, associated with enhanced Type I IFN response, was found after intratumoral administration of c-di-GMP. As an adjuvant, c-di-GMP also boosted OVA peptide vaccination [35]. The intravenous administration of c-di-GMP, encapsulated in YSK05-liposomes, into mice significantly induced Type I IFN production and activation of natural killer (NK) cells. This resulted in a strong antitumor effect in a lung metastasis mouse model [47]. STING activation by 3′,3′-cGAMP in chronic lymphocytic leukemia model caused apoptosis induction and tumor regression. A similar effect was also seen in syngeneic or immunodeficient mice, grafted with multiple myeloma. This report indicated the potential of CDNs in direct eradication of malignant B cells [48].

### 4.2. Targeting Non-Canonical Mammalian CDN and Analogs

The recent report, showing particular human STING varients refractory to bacterial CDNs (with canonical 3′–5′ linkages), suggests that non-canonical mammalian CDNs (2′–5′ linkages) are the preferred set of compounds for advancement to clinical trials [49]. In melanoma and colon cancer model, intratumoral injection of 2′,3′-cGAMP stimulated CD8+ T cell response, delayed injected tumor growth and induced systemic anti-tumor immune response. The antitumor potential of 2′,3′-cGAMP was further enhanced by the blockade of both PD1 and CTLA4 [50]. TLR9 agonist and 2′,3′-cGAMP were also shown to synergistically induce innate and adaptive immune response. The combination of TLR9 agonist and 2′,3′-cGAMP induced strong Th1-type responses and cytotoxic CD8+ T-cell responses. Intratumoral injection of TLR9 agonist and 2′,3′-cGAMP reduced tumor size in mouse melanoma model [51]. 2′,3′-cGAMP was also shown to have significant anti-tumor effect in adenocarcinoma model where intratumoral injection of this STING agonist enhanced cytokine production, triggered dendritic cell activation and selectively activated apoptosis in tumor cells. It was also shown that 2′,3′-cGAMP treatment enhanced the expression of STING and IRF3. This amplification loop seemed to have positively influenced the anti-tumor effect. Combination therapy of 2′,3′-cGAMP and the DNA damaging chemotherapeutic drug 5-fluorouracil (5-FU) not only showed synergistic anti-tumor effects but combination of 2′,3′-cGAMP and low doses of 5-FU also reduced adverse toxicity of 5-FU chemotherapy [11]. Exogenous 2′,3′-cGAMP treatment and radiation were also shown to synergistically amplify anti-tumor immune response [12].

The successful application of non-canonical 2′,3′-cGAMP in eliciting anti-tumor immunity, paved the way for exploring the anti-cancer immunostimulatory potential of chemically synthesized non-canonical CDN analogs. Rationally designed synthetic dithio mixed-linkage CDNs were shown to potentially activate the five known human STING variants. ML RR-S2 CDA, the lead compound, having enhanced stability and lipophilicity, showed improved STING activation and anti-cancer potential both in vitro and in vivo [42]. Strong significant tumor regression in the B16 melanoma model, CT26 colon cancer model, or the 4T1 breast cancer was seen after intratumoral injection of the synthetic CDN. Robust systemic antigen-specific CD8+ T cell response was also induced causing rejection of distant, non-injected tumors. Around 50% of treated animals were tumor-free and had more than 150 days of survival after intratumoral injection. Synthetic CDN also conferred absolute protection against tumor re-challenge by providing long-lived immunologic memory. Though intratumoral injection of synthetic CDNs can limit their application, the abscopal response can potentially activate a strong systemic immune response [40].

The promising synergistic anti-tumor effect of 2′,3′-cGAMP and radiation [12] has also motivated researchers to optimize the therapeutic potential of synthetic CDN and radiation combination therapy. R_P_, R_P_ dithio 2′,3′-CDN molecules combined with CT-guided radiotherapy showed synergistic anti-cancer immune response in local and distal tumors in a murine pancreatic cancer model. This synergistic effect produces a two-phase response, where the initial TNFα secretion driven T-cell–independent hemorrhagic necrosis is followed by a CD+ 8 T-cell–dependent recurrence control [52]. Synthetic 2′,3′-cGAMP analogs are also being explored as vaccine adjuvants in cancer immunotherapy. Cellular cancer vaccine, STINGVAX, was synthesized by combining synthetic 2′,3′ CDNs with granulocyte-macrophage colony-stimulating factor (GMCSF). STINGVAX showed strong in vivo antitumor efficacy in multiple cancer therapeutic models. Rationally designed synthetic 2′,3′ CDNs, e.g., one with a R_P_, R_P_ dithio diastereomer and another with a non-canonical 2′,3′ mixed linkage (c [A (2′,5′) pA (3′,5′) p]), boosted antitumor efficacy of STINGVAX in multiple aggressive cancer therapeutic models. Interestingly, in comparison to murine cells, where R_P_, R_P_ dithio 2′,3′ CDN molecules were shown to be most potent STING stimulator in vivo, synthetic CDNs containing 2′,3′ mix linkage phosphate bridge seemed to be more potent activators of human APCs. Significant PD-L1 up regulation associated with tumor infiltrating CD8+ T cells was found in tumors from STINGVAX treated mice. Combination of STINGVAX and PD-1 blockage could target poorly immunogenic tumors that were totally unresponsive to PD-1 blockage [53].

Successful application of synthetic CDN analogs in eliciting anti-tumor immune response via STING activation allows us to explore additional alternate avenues to therapeutically target this pathway. Small molecule and/or modified nucleic acid activators of cGAS can be an alternative along the line. One advantage of activating the enzyme cGAS over STING is that this upstream enzyme can produce an amplified signal relative to the STING receptor-small molecule interaction. In addition, structural similarity between human and mouse cGAS would also be advantageous in extrapolating mouse model results to clinical trials. Along this line, Hall et al. have very recently reported a high affinity cGAS inhibitor which can be tested for its therapeutic application in context of cancer or autoimmune disorder [54].

### 5. cGAS/STING Pathway in Carcinogenesis

Although cGAS/STING pathway is being targeted for potential cancer immune therapy, evidence indicates that cGAS/STING pathway contributes to inflammatory carcinogenesis as well. Severe side effects, including autoimmune and inflammatory response and direct tissue toxicity, limit the anti-tumor potential of Type I IFN therapy and cGAS/STING signaling modulation therapy.

STING was shown to enhance inflammatory cytokine levels of infiltrating phagocytes and thereby promote inflammation driven carcinogenesis. STING−/− mice were resistant to mutagen (DMBA) induced skin carcinoma compared to wild-type mice [30]. DNA sensing via cGAS/STING pathway also induces tolerogenic response in mice [55] by activating immune regulatory mechanisms. Indoleamine 2,3 dioxygenase (IDO) is an important immune check point and STING induced IDO activation in tumor microenvironment was shown to promote the growth of Lewis lung carcinoma (LLC). STING ablation enhanced CD8+ T cell infiltration, tumor cell killing, decreased suppressor cell infiltration and IL-10 production in tumor microenvironment in LLC mice model, indicating the role STING signaling in attenuation of CD8+ T cell functions during tumorigenesis [56]. Molecular insight of virus induced carcinogenesis has also shed light on the importance of cGAS/STING pathway in this context. Expression of STING was shown to be up regulated and activated in HPV+ tongue squamous cell carcinoma (TSCC) samples and activated STING promoted the induction of several immunosuppressive cytokines and chemokines, e.g., IL-10, CCL22, etc., that facilitated regulatory T cells (Tregs) infiltration and thereby helped in carcinogenesis by affecting anti-cancer immune response [57].

Recent evidence suggests that self-DNA induced activation of cGAS/STING pathway is responsible for autoimmune and inflammatory disorders [8,15,16]. Given the genetic stress, DNA damage and nuclear DNA leakage in cancer, cGAS/STING activation can potentially help in inflammation induced carcinogenesis.

It should also be mentioned that the carcinogenic effect of cGAS/STING signaling may be cancer type specific. While STING−/− mice were resistant to DMBA induced skin carcinoma [54], STING−/− mice developed colonic tumor at an enhanced frequency compared to WT mice [58]. Thus, tumor type, location and tumor microenvironment may play significant role in dictating the anti-cancer or carcinogenic role of cGAS/STING pathway.

## 6. Concluding Remarks

cGAS/STING pathway seems to be a double-edged sword in cancer, and hence it is important to understand the molecular details and spatio-temporal regulation of this pathway in the context of cancer. Understanding how to shift this balance towards anti-cancer immune activation represents an attractive therapeutic strategy to combat cancer. It is logical to assume that the concentration of STING activators, such as 2′,3′-cGAMP and other synthetic CDN analogs, have key roles to play in this context. While optimal CDN concentration helps in eliciting anti-tumor immune response, high CDN level, followed by uncontrolled STING activation, may lead to sustained inflammation and carcinogenesis. Thus, determining optimal 2′,3′-cGAMP level is of extreme importance in context of cancer therapeutics. We have reported a RNA based fluorescent biosensor against 2′,3′-cGAMP [59]. The ability to measure 2′,3′-cGAMP levels in cells will impact our understanding about the optimal activation of cGAS that can elicit a robust anti-cancer immune response, while still not activating inflammation induced carcinogenesis. The biosensor can also be used in high throughput format to screen for small molecule modulators of cGAS activity in cancer therapeutic perspective. Measuring cGAS activity will also help us identifying DCs in tumor microenvironments that are CDN sensitive and therefore may help in effective therapeutics.

cGAS/STING pathway has the potential to be targeted for effective cancer therapeutics. A deeper knowledge of cGAS/STING pathway and its regulation could point towards successful therapeutic targeting.
